# A critical review of PASBio's argument structures for biomedical verbs

**DOI:** 10.1186/1471-2105-7-S3-S5

**Published:** 2006-11-24

**Authors:** K Bretonnel Cohen, Lawrence Hunter

**Affiliations:** 1 Center for Computational Pharmacology, University of Colorado School of Medicine, Aurora, CO, USA; 2 Dept of Linguistics, University of Colorado at Boulder, Colorado, USA

## Abstract

**Background:**

Propositional representations of biomedical knowledge are a critical component of most aspects of semantic mining in biomedicine. However, the proper set of propositions has yet to be determined. Recently, the PASBio project proposed a set of propositions and argument structures for biomedical verbs. This initial set of representations presents an opportunity for evaluating the suitability of predicate-argument structures as a scheme for representing verbal semantics in the biomedical domain. Here, we quantitatively evaluate several dimensions of the initial PASBio propositional structure repository.

**Results:**

We propose a number of metrics and heuristics related to arity, role labelling, argument realization, and corpus coverage for evaluating large-scale predicate-argument structure proposals. We evaluate the metrics and heuristics by applying them to PASBio 1.0.

**Conclusion:**

PASBio demonstrates the suitability of predicate-argument structures for representing aspects of the semantics of biomedical verbs. Metrics related to *theta*-criterion violations and to the distribution of arguments are able to detect flaws in semantic representations, given a set of predicate-argument structures and a relatively small corpus annotated with them.

## Background

### Semantic representation in biomedicine: the current state of the art

Most tasks related to semantic mining in biomedicine, from manual annotation of experimental data to information extraction from free text, depend critically on a target semantic representation of the domain. Unfortunately, no generally accepted standard for such a representation yet exists. This problem is a microcosm of the larger problem that bedevils general semantic processing: the fact that biomedicine forms a sublanguage of general English ([1], [2], [3], [4] and [5]) may reduce the scale of the problem, but it does not change the type of the problem. Simply put, what is an optimal (or even adequate) set of propositions for representing the semantics of biomedical verbs?

Although related to the question of a proper ontology for representing biomedical knowledge, the propositional representation question addresses the set of relationships that link fundamental ontological elements into the assertions that are the essence of biomedical discourse. Propositional representations are usually associated with verbs (e.g. "inhibits"), although nominalized forms of these propositions (e.g. "inhibition") clearly play an important (and possibly dominant – see Friedman et al. [4] and Tateisi et al. [6]) role in biomedical texts. Propositional representation schemes specify the particular types of relationships (that is, the propositions), along with the number and type of related entities (that is, the arguments of the proposition). The representation may also specify allowable modifiers of propositions (e.g. temporal or spatial localizations). These are "content theories," specifying not only the form of a proper representation, but specific predicates, arguments and restrictions necessary and sufficient to capture meaning in biomedicine.

Large-scale projects to develop propositional representation schemes (e.g. FrameNet [7]) and to create gold-standard proposition-labelled text corpora (e.g. PropBank [8]) have had salutary effects on the ability of computational systems to do semantic mining in general English (as well as in other natural languages, including Chinese and Korean). Biomedical concepts and language are different enough from "newswire" domains that existing propositional representations are inadequate for capturing biomedical knowledge.

Recently, the PASBio project [9] released a set of propositional representations for a small set of biomedically relevant verbs. PASBio is similar in method to FrameNet, and in goals to PropBank. The utility of the initial representational scheme is seen in its application to LSAT, a system that extracted more than 4,000 complex propositions about alternative splicing of mammalian genes from more than 14,000 PubMed abstracts [10]. Furthermore, the project's methodology has recently been successfully extended to clinical texts [11]. Although the PASBio project is not unique, most prior work (e.g. Tateisi et al. (2004)[6]) is now moribund, highlighting the potential difficulties of work in this area. A second molecular-biology-oriented proposition bank, BioProp [12], is described below in Section 6.2 entitled *PASBio versus BioProp.*

### Evaluation of semantic representations

These successful applications make this the appropriate time to raise the question of whether the PASBio method and its specific representational scheme are optimal (or even adequate) for the general problem of representation of molecular biology concepts. The initial release of PASBio explicitly recognizes its incompleteness, so the critical questions are about whether it is structured properly, has appropriate content, and how much additional work would be required to make it appropriately broad. Our approach to addressing these questions is modelled on Baker and Ruppenhofer's comparison of FrameNet [13] and the Levin verb classes, as well as on Baker et al. [14].

A proposed representational scheme can be evaluated in many ways, but quantitative measures that reflect specific desired characteristics of any scheme are particularly attractive. Although the mapping to the desirable characteristics may be partial, their quantitative nature obviates potential concerns about theoretical biases. We therefore provide quantitative data on the following: the distribution of arity of argument sets, distribution of thematic role types versus individual thematic roles in the argument sets, violations of the *θ*-criterion, coverage of the verbs in two biomedical corpora, and distribution of arguments in the example data.

The richness of a representation can be quantified by exploring the arity of its argument sets. When they address propositions at all, most previous biomedical information extraction systems have targeted binary relations. As Rzhetsky et al. [15], McDonald et al. [16], and others have pointed out, many biomedical relations are in fact of greater than binary arity. A representational system that points us towards greater than binary relations has the potential to stimulate a qualitative advance in biomedical information extraction.

There is a potential mapping between propositional representations and frame-based representations (in the sense of Minsky [17], e.g. such as could be well-handled by the Protégé frame system). However, the distribution of thematic roles (or "slot" types in a frame representation) can be either drawn from a narrow list of generic thematic role types (e.g. *Agent, Theme*, and *Goal*), or from individual thematic roles – specialized roles particular to a few (or a single) frames (e.g. the *translation product, translation source*, and *translation location *of the biomedical verb *translate *– see [18], p. 550). In addition to providing information relevant to the design of an efficient formal representation scheme (frame in the Minskian sense), it also has implications for the types of data that need to be gathered in order to specify the correct roles and their relationships to texts (i.e., frames in the sense of FrameNet [7] op cit.). There is generally thought to be only a small set of thematic role types, some subset of which characterizes the arguments of every verb. They reflect deep semantic relations, such as causation and volition. There is little agreement on either the labels or the numbers of thematic role types. In contrast, the number of individual thematic roles is unbounded, and there are few similarities in them across verbs. They reflect only shallow semantic relations, and their labels are essentially arbitrary. Thematic role types capture many linguistic generalizations, but it is difficult to get agreement on their actual use in representations. Individual thematic roles miss many linguistic generalizations, but it is easier to get agreement on them in representations (and perhaps in annotation), and they do capture some *semantic *generalizations. (Note also that it is possible to mix them in representations, e.g. using the thematic role type of *agent *for one argument of a verb, and individual thematic roles for the others). Furthermore, thematic roles could themselves be productively represented within a hierarchy, in the style of the OBO Relation Hierarchy [19]. For example, individual thematic roles can be represented as leaf nodes, thematic role types as top-level superclasses, and intervening nodes expressing intermediate levels of abstraction.

Given representations with particular arities and role choices, we would like quantitative measures of whether or not they are correct. We approached this by looking at the applications of the PASBio predicate-argument structures (PASs) to the example sentences that are distributed with the representations themselves. We examined these for violations of the *θ*-criterion, and for distributional characteristics of the arguments.

As stated by Dowty [18], following work by Chomsky, the *θ*-criterion (or similar principles such as the Argument Realization Principle in non-GB frameworks, e.g. Goldberg (2005)[20]) includes the claim that "the same *θ*-role is not assigned to two NP arguments of the same predicate" ([20] p. 549). Work in non-GB frameworks such as Fillmore [21] makes similar assumptions. In particular, the *θ*-criterion should hold in cases where individual thematic roles are used in representations rather than thematic role types (op. cit., p. 550). So, quantifying the number of times that the PASBio representations led to *θ*-criterion violations in their example sentences (see Table 4) is a quantifiable and non-subjective way of assessing the fit of the representations to at least a small sample (equal to the number of illustrative sentences in PASBio) of molecular biology texts. Note that the NomBank project uses a similar heuristic to detect annotation errors – they examine annotator output for multiple instances of the same argument role [22].

We also experimented with using distributional characteristics of arguments as a heuristic for detecting invalid PAS representations. We manually examined all PASBio verb representations for arguments that were in complementary distribution with each other in the example sentences, i.e. situations where some argument Arg*i *never appears with Arg*j*. Where complementary distribution exists, one might suspect that either the two arguments should be combined, or the predicate should be split in two, with one predicate taking Arg*i *and the other taking Arg*j*.

The arity, role choice, *θ*-criterion violation and argument distribution evaluations are informative with respect to how "good" PASBio's argument structures are for semantic representation in biomedicine. The corpus coverage evaluations are relevant to the question of the amount of work yet to be done if the PASBio approach is to be adopted for biomedical text in general: assessment of the proportion of verbs in biomedical texts that are covered by PASBio offers an indication of how well the representational approach will scale to realistic problems.

### PASBio in context

The remainder of this paper is devoted to quantitative analysis of PASBio; here, brief qualitative comparisons to related resources are given.

#### PASBio versus NLM's Semantic Network representation

The US National Library of Medicine also provides a general representation of biomedical verb semantics: the NLM Semantic Network [23]. Since the NLM Semantic Network has not been used to annotate a corpus as PASBio has, it is not currently possible to make the same quantitative comparisons with the Semantic Network.

The Semantic Network groups verbs via a troponomy-like relation, labelled isa. For example, *treats *isa *affects, affects *isa *functionally-related-to*, and *functionally-related-to *isa *associated-with*. Verbs have binary argument sets, defined in terms of semantic classes. Practical application of the Semantic Network depends, of course, not just on recognizing the relevant verbs, but on the ability to recognize and map to a wide variety of semantic classes of arguments. For a system that actually does so, see [24].

In the Semantic Network approach to representing verb semantics, arguments are binary, and are limited to specific semantic classes in the Semantic Network ontology. In contrast, PASBio arguments are not limited with respect to arity, and there are only broad restrictions on argument instantiations. Table 1 shows the arguments of *transcribe*.

#### PASBio versus BioProp

Recent papers by Tsai et al [12] and Chou et al [25]. reported on the construction of BioProp, a proposition bank built on top of the 500 syntactically parsed abstracts currently available in the GENIA corpus. The project involved annotating the arguments of 30 frequent biomedical verbs, using PropBank PASs to the greatest extent possible, and defining new PASs for verbs that are not present in PropBank at all. BioProp adheres to the PropBank distinction between adjuncts and core arguments. Like PASBio, and unlike the Semantic Network, the arity of BioProp PASs is unrestricted. Since neither the actual set of BioProp PASs nor the annotated corpus itself have been made publicly available, it was not possible to make the same quantitative assessments of the BioProp PASs as we did for PASBio.

## Results and Discussion

### Arity

Table 2 shows the distribution of PASBio predicates across binary, ternary, and larger arities. 65% (22/34) of the PASBio predicates have greater than binary arity.

### Roles

Only a single predicate uses thematic role types in its argument representation. *Block *has an agent and a theme as its arguments. For all other predicates, they may have an agent, but all other arguments are individual thematic roles.

### Overlap with verbs in the corpora

Table 3 shows the percentages of verb tokens in the corpora that are covered by the verbs in PASBio. (When a PASBio verb represents multiple predicates, there is no way to determine from the annotations of the corpora which predicate(s) are represented, so we back off to the verbs themselves.) Overlap with the verbs in the corpora was not large, ranging from a low of 4.9% for BioIE-Oncology to a high of 12.1% for BioIE-CYP450.

Type-level overlap is of course quite small: 28/871 for BioIE (one verb, *splice*, does not occur at all in BioIE); 24/649 for BioIE-CYP450 *(delete, disrupt, proliferate, skip *and *splice *do not appear in BioIE-CYP450); 26/601 for BioIE-Oncology *(eliminate, splice*, and *translate *do not appear in BioIE-Oncology); and 28/1077 for GENIA *(skip *does not appear in GENIA). (These numbers actually underestimate type-level coverage somewhat. Our type counts are based on stems, rather than lemmas, so e.g. *bind *and *bound *count as two types, rather than one; since the numerators are so swamped by the denominators, we did not make this correction.)

### Violations of the *θ*-criterion

We only found a single verb whose example sentences violated the *θ*-criterion. Table 4 gives the representation of the predicate *express. 01 *in PASBio 1.0, along with the specific examples (three out of fifteen) that violate the criterion. (We discuss alternative analyses of this data at some length in the *Results of the evaluation *section.)

### Argument distributions in example sentences

Examining the examples for *inhibit. 01*, we noted that Arg1 (entity being inhibited) and Arg2 (process being inhibited) never coöccurred. (They appear to coöccur in two examples in Rev. 1.0, but these turned out to be annotation errors.) On the basis of this observation, the PASBio project will be combining these into a single argument in the next release. In the case of the examples for *truncate*, we noticed that Arg1 and Arg2 never coöccurred (again, once a single annotation error was corrected). This led to a decision by the PASBio project to split *truncate *into two predicates in the next release. The examples for *splice.01 *contain multiple pairs of non-coöccurring arguments, but none of them obviously indicated erroneous representations.

## Discussion

### Results of the evaluation

The PASBio project compares itself at some length to both FrameNet and PropBank, and describes itself explicitly as an attempt at building a PropBank-like resource. However, to some extent the current revision combines the worst attributes of both FrameNet and PropBank. Like FrameNet, its "corpus" data consists of only a small number of illustrative sentences. (In contrast, PropBank commits to tagging every instance of every (verbal) predicator.) Like PropBank (and unlike the Semantic Network), its representations are purely lexical, with no higher level of organization.

This characterization is in some sense unfair to both PASBio and PropBank, since both projects hope to eventually incorporate FrameNet-compatible representations; it is doubly unfair to PASBio, which hopes to add a considerably more comprehensive set of examples. However, this characterization *does *support the value of adding such work to PASBio, and by implication the value of funding such work.

65% of the PASBio predicates have greater than binary arity. Our *θ*-criterion violation and distributional analyses suggest that on the whole, these greater-than-binary arities are appropriate. As Rzhetsky et al. [15] and others have pointed out, most biomedical information extraction systems have limited themselves to relations of binary arity, but many biomedical relations are of greater than binary arity. The predominance of greater-than-binary relations in PASBio suggests that its representations have the potential to stimulate a qualitative advance in biomedical information extraction.

The role-labelling choices in PASBio are encouraging, as well. Their individual thematic roles facilitate mapping from predicates to higher-level frames, and should facilitate rapid corpus annotation, as well. Addition of thematic role types to the individual thematic roles may aid in leveraging syntactic information, but the current choice is sensible. We return to the issue of roles below.

Wattarujeekrit et al. pointed out that only 6 of 29 PASBio verbs had the same sense and same structure as the corresponding PropBank verbs (p. 12). This finding underscores the necessity of investing in the construction of NLP resources that are tailored to the biomedical domain. (The extent to which this requires de novo construction, versus lexical tuning (see e.g.[25]) of preëxisting resources, is a question worth serious investigation; the PASBio and BioProp projects both are relevant sources of data for answering it.) We compared the set of PASBio verbs with the set of verbs indexed in VerbNet 2.0. This resource expands on the original set of verbs in Levin [26]. (Note that this comparison involves verbs, not predicates – e.g., the predicates *translate.01, .02*, and *.03 *are collapsed here into a single "verb.") We accepted any homograph as a match; since some of the verbs are truly polysemous, this yields an overestimate of the representation of PASBio verbs in VerbNet. On this measure, 79% of PASBio verbs (23/29) have homographs in VerbNet. Levin classes lay some of the groundwork for understanding how syntactic form is related to propositional meaning, so this is an encouraging finding, suggesting that some of that groundwork may be done. A more fine-grained assessment of the extent to which those homographs represent the same verb meanings as PASBio, and more importantly, the same meanings as the common verbs in the corpora – e.g., *express *almost certainly does not – remains for future work.

A major goal of this paper was to find metrics that would let us gauge the *quality *of the representations and their suitability for representing actual textual data that would be both quantifiable, and not prone to (our own) theoretical biases and assumptions. The screening for *θ*-criterion violations and for arguments in complementary distribution are our suggestions for such metrics. The low incidence of *θ*-criterion violations is suggestive of good-quality representations. We found *θ*-criterion violations only for a single verb (see Table 4). There are actually a number of alternative ways of looking at this data. For example, if one assumes a dependency parse, rather than the Penn-Treebank-style parse that we *did *assume, then in all three of the *θ*-criterion violations shown in Table 4, the *cell surface *mentions at the end of all three sentences would be dependent on the *cell *mentions at the beginning of each sentence. This would remove all three *θ*-criterion violations – a nice result for PASBio's representations, but one which would certainly call the probative value of the *θ*-criterion violation metric into question. Alternatively, one might use the first and third examples to suggest an alternative representation in which there is an Arg0 that is the agent of expression, in which case the *cell surface *mentions could be treated either as Arg3s or as ARG-LOCs without causing a *θ*-criterion violation. However, in a larger set of examples, it becomes clear that it is difficult on semantic grounds to justify the assignment of *cells *to an agentive role for this verb. A third approach would be to preserve the distinction between adjuncts and core arguments, rather than treating all arguments as core arguments – there would then be no *θ*-criterion violations here, since presumably most of the problematic constituents would be labelled as adjunctive ARGM-LOCs and by virtue of their adjunctive status would be under no theoretical limits as to number of instances. However, this would ignore one of the crucial claims of the PASBio project (and a finding from our own work with domain experts), which is precisely that with the exception of negative elements in text, knowledge representation in this domain requires that we *not *make a distinction between adjuncts and core arguments. Note also that blurring the distinction  between arguments and adjuncts was one of the motivations for using  the higher-numbered arguments in PropBank [8]. Our analysis here need assume only a relatively non-controversial syntactic analysis of the examples, a semantically appropriate analysis of agency for this verb, and the non-applicability of the core/adjunct argument distinction for this domain, so we note again the utility of a similar heuristic in the NomBank project (op cit) and continue to propose the utility of the *θ*-criterion for evaluating PAS proposals. We note also that  there is a useful reason for maintaining the argument/adjunct  distinction: adjuncts are equally centrally important to many  different events, and from a machine learning perspective, it is  desirable to be able to count them as "the same" over the entire data  set.  However, from an applications perspective, it seems more  desirable to maintain biological integrity in the knowledge  representation than to surrender it in exchange for a higher  performance number on a machine learning task. Recent work by Merlo and Ferrer [31] points out well-argued distributional and theoretical reasons for maintaining the argument/adjunct distinction, distinct from the strictly utilitarian reasons that we argue against.

### Suggestions for future directions

In the work reported here, the PASs were evaluated using the annotated data that is provided with them by the PASBio project. This was a deliberate methodological choice – it allowed us to evaluate the metrics proposed in this paper using annotations that were not produced by us, and also allowed us to investigate the adequacy of small data sets for detection of representational flaws. However, as one reviewer pointed out, a fuller evaluation of this specific set of representations would require using the proposed PAS to annotate a sample of biological texts that were not used to develop the representations. We are currently pursuing such a project, in the course of which we are evaluating not only the PASBio representations, but also those provided by the BioProp, PropBank, and other resources.

The predicate-specific problems that we uncovered by looking at *θ*-criterion violations and complementary distributions of arguments reflect a fundamental representational issue that is not frequently addressed (and occasionally misunderstood) in the literature on biomedical information extraction. This is the contrast between the different goals and different scopes of representations for information extraction, and representations of the semantics of verbs.

The best-known model for representations for information extraction is that of the MUCs. These representations were frame-based, and large-large enough that it was unlikely that the slot-fillers would all be arguments of a single verb.

Shallow representations of the semantics of verbs require a model of what arguments the verb can take. An argument is a syntactic constituent (e.g. noun phrase) to which a role label (e.g. Arg0, Arg1, Arg-LOC) is assigned. These representations are smaller than MUC-type frames, since they are by definition restricted to a single verb. When the PASBio data represents the length of a transcript as an argument of *express*, it is failing to distinguish between the predicate-argument structure of the verb *express *and the slots of an expression frame. We argue in the introduction that frame-like representations are desirable for biomedical information extraction, but it is important to maintain this distinction between verb-level and more abstract representations: it gives us access to a well-understood and constrained formalism, provides a handle on role-labelling-like formulations of the semantic analysis task, and facilitates the annotation of corpora, without losing the expressive power of event-based representations.

With only 29 verbs (representing 34 predicates) in the current revision, our data on overlap with the corpora suggests (not surprisingly) that the current revision is far too small. However, a relatively small number of additions would increase coverage enormously. Version 1.0 of PASBio used a model of the domain, rather than frequency, to motivate verb choices. For the second version, switching to frequency data seems called for. Lessons learned from the PropBank project also suggest migrating from the FrameNet-like set of isolated sentences in the current revision to a fully-developed, treebanked corpus. PASBIO 1.0 represents 29 verbs chosen for their use in the description of gene expression and related events; Table 5 gives the percentage of coverage, and counts of tokens, in the corpora that could be covered by choosing instead the 29 most frequent verbs (with some filtering of non-biomedical verbs).

As Wattarujeekrit et al. point out, there is a natural role for ontologies in constraining the arguments for biomedical PAS. The Semantic Network uses the UMLS in this way; the Gene Ontology and other OBO projects seem likely candidates for PASBio, but no current biomedical verb representation project has moved in this direction yet. As of yet, PASBio has not followed up on this insight; we suggest that this is the next big step for PASBio or similar projects. Lu et al. (unpublished data) reports on a pilot data annotation and information extraction project whose results are compatible with the hypothesis that using ontologies to constrain the slot-fillers of complex, PASBio-like high-arity predicate-argument structures is possible for two tasks: annotation of natural-language texts in restricted domains, and production of information extraction systems. Lu et al. produced a completely ontology-driven corpus and relation extraction system. The ontology was specially built for this project, but it was constrained to be a subset of third-party ontologies and other data sources: the Gene Ontology provided the elements of a cellular component ontology and of an ontology of protein transport events, and the Entrez Gene database was used as the source for all protein annotations. These data sources provided the ontology with reference to which the corpus was annotated, and also provided the reference knowledge model to which their information extraction system mapped its outputs. The event ontology was then enhanced with linguistic patterns based on a PASBio-like representation of predicate-argument structure. They achieved high inter-annotator agreement rates on the annotation task and competitive performance on the information extraction task, demonstrating that ontology-constrained PAS are practical both as a model for corpus annotation and as the organizing principle of biomedical information extraction systems.

The current version of PASBio takes definitions from WordNet. In view of the demonstrable problems in mapping to WordNet senses [14] and the high overlap between PASBio and VerbNet 2.0, we suggest a change to VerbNet. This would also move PASBio towards its desired frame-like organizational structure.

## Conclusion

Our findings support the hypothesis that predicate-argument structures, as illustrated by the PASBio project, are a viable formalism for building shallow semantic representations of biomedical verbs. They leave unrepresented many important aspects of verbal semantics – aspect, manner, causation, to mention just a few – but they provide an important handle on the problem, and one that is salutary both for the corpus construction efforts and for the approaches to NLP that have allowed for rapid progress in General English domains.

Our results also demonstrate that given a set of predicate-argument structures and a data set annotated with respect to that set of PASs, the *θ*-criterion violation and complementary distribution metrics were both effective at finding flaws in the proposed set of representations. The amount of annotated data that would be optimally efficient for uncovering such flaws has not been investigated here, but the data presented here indicates that a rather small amount of data – as few as ten annotated sentences – is sufficient to uncover at least some representational issues.

## Methods

### Materials

We used releases 1.0 of PASBio, 0.9 of the BioIE corpus [27], 3.0p of the GENIA corpus [28], and 2.0 of VerbNet [29]. The BioIE corpus has two separate subsections, one dealing with CYP450 and the other with oncology. We refer to the entire corpus as BioIE, to the CYP450 section as BioIE-CYP450, and to the oncology section as BioIE-Oncology.

### Determining verb frequencies

We extracted all verb tokens from both corpora by using egrep to search for tokens whose tags matched the pattern VB.? in the BioIE. mrg files and the GENIA GENIAcorpus3.02.pos.txt file. (This is a potential source of a small amount of noise in the BioIE data, since not all POS tags are curated in that data. Fifty tokens from the BioIE data, including numerals, punctuation marks, and single letters, were clearly mis-tagged as verbs. We excluded them from the analysis.) We then collapsed inflected forms of verbs by applying the Porter stemming algorithm [30], using a publicly available implementation from the Tartarus web site.

### Violations of the *θ*-criterion

To detect violations of the *θ*-criterion, we manually looked for example sentences that contained one or more semantic arguments with more than one syntactic constituent. To avoid theory-specific differences in syntactic parse structures for complex NPs, we only counted clearly discontinuous constituents.

### Argument distributions in example sentences

We manually examined all PASBio verb representations for arguments that were in complementary distribution with each other in the example sentences, i.e. situations where some argument Arg*i *never appears with Arg*j*.

## Figures and Tables

**Table 1:** Arguments of *transcribe *in PASBio 1.0.

**Arg0**	causer, agent (Comment: protein)
**Arg1**	entity transcribed (Comment: gene, DNA)
**Arg2**	transcription site (Comment: promoter)
**Arg3**	entity after transcription
**Arg4**	location as organ or tissue

**Table 2:** Arity of PASBio predicates. The column headed *2 *lists all predicates with two arguments, the column headed *5 *lists all predicates with five arguments, etc.

2	3	4	5
*abolish*	*confer*	*modify*	*alter*
*begin.01*	*decrease*	*mutate*	*initiate*
*begin.02*	*delete*	*splice.01*	*transcribe*
*block*	*develop*	*splice.02*	
*catalyze*	*disrupt*	*truncate*	
*encode*	*eliminate*		
*generate*	*express*		
*lead*	*inhibit*		
*recognize*	*lose*		
*result*	*proliferate*		
*transform.02*	*skip*		
*translate.03*	*transform.01*		
	*translate.01*		
	*translate.02*		

**Table 3:** Overlap between PASBio and the corpora. For each corpus, we give the percentage of verb tokens that could be accounted for by the PASBio verbs. The *Verb tokens *column gives the number of tokens covered by PASBio/the total number of verb tokens in the corpus. The *Verb types *column gives the number of types covered by PASBio/the total number of verb types in the corpus. See the text for why the numerator in the latter is not always 29.

Corpus	Verb tokens	Verb types
BioIE (both)	8.8% (1509/17,186)	3.2% (28/871)
BioIE-P450	12.1% (1,148/9,455)	3.7% (24/649)
BioIE-Onc	4.9% (379/7,731)	4.3% (26/601)
GENIA	8.5% (4,416/51,879)	2.6% (28/1077)

**Table 4:** Representation of *express *in PASBio 1.0. The first three lines give the argument structure. The last three lines give the three examples that violate the θ-criterion: underlined phrases are mapped to Arg3.

Arg	mnemonic
**Arg1**	named entity being expressed (gene or gene products)
**Arg2**	property of the existing named entity
**Arg3**	location referring to organelle, cell or tissue
Example number	Example text
**PNAS 2**	T cells from double TCR transgenic mice express only one or the other of the two available TCRs at the cell surface.
**PNAS 4**	In such cells, two in-frame α transcripts are produced, but only one is expressed at the cell surface.
**PNAS 5**	T cells in mice carrying transgenes encoding TCR α and β proteins predominantly express the transgene-encoded TRC proteins at the cell surface.

**Table 5:** Verb tokens covered by the 29 most frequent verbs in each corpus. These counts reflect filtering some non-biomedical verbs, such as *be*. Compare these data to those in Table 3.

Corpus	Percentage	Tokens
BioIE (both)	23.8%	4,088/17,186
BioIE-P450	29.2%	2,757/9,455
BioIE-Oncology	21.7%	1,675/7,731
GENIA	29.6%	15,363/51,879
